# Transcription factor ZNF148 is a negative regulator of human muscle differentiation

**DOI:** 10.1038/s41598-017-08267-5

**Published:** 2017-08-15

**Authors:** Jesse Bakke, William C. Wright, Anthony E. Zamora, Su Sien Ong, Yue-Ming Wang, Jessica D. Hoyer, Christopher T. Brewer, Paul G. Thomas, Taosheng Chen

**Affiliations:** 1 Department of Chemical Biology and Therapeutics, St. Jude Children’s Research Hospital, Memphis, Tennessee USA; 2 Integrated Biomedical Sciences Program, University of Tennessee Health Science Center, Memphis, Tennessee USA; 3 Department of Immunology, St. Jude Children’s Research Hospital, Memphis, Tennessee USA

## Abstract

Muscle differentiation is a complex process in which muscle progenitor cells undergo determination and eventually cellular fusion. This process is heavily regulated by such master transcription factors as MYOD and members of the MEF2 family. Here, we show that the transcription factor ZNF148 plays a direct role in human muscle cell differentiation. Downregulation of ZNF148 drives the formation of a muscle phenotype with rapid expression of myosin heavy chain, even in proliferative conditions. This phenotype was most likely mediated by the robust and swift upregulation of MYOD and MEF2C.

## Introduction

Muscle cell differentiation occurs in a series of tightly coordinated events in which myoblasts differentiate in three specific stages. In the first stage, myoblasts exit the cell cycle and initiate muscle-specific gene expression. In the second stage, myoblasts align with one another in a cell type–specific manner. The final stage of myoblast differentiation comprises the events of cell fusion, in which many adjacently aligned myoblasts establish connections with one another and form large multinucleated cells^1, 2^. Progression through these stage is extensively coordinated by the basic helix-loop-helix proteins myogenic regulatory factor 4^3^, myogenic determination factor (MYOD)^4^, myogenic factor 5^5^, and myogenin, in addition to the myocyte enhancer factor 2 (MEF2) family^6^. Briefly, MYOD and myogenic factor 5 drive the determination of progenitor cells into myoblasts. Myogenin then promotes differentiation of the myoblasts to form myotubes^7^. The MEF2 family members (MEF2A–D), which are extensively but not entirely redundant^8^, act in a cooperative manner with the basic helix-loop-helix proteins to potentiate muscle differentiation^7^. Thus, these two protein groups cooperate to promote terminal myotube formation. The dynamic regulation imparted by their cooperation can be recapitulated *in vitro*, particularly in the mouse C2C12 cell line^9^ and the human LHCN-M2 cell line^10^.

The Kruppel-like zinc finger protein 148 (ZNF148), also known as β enolase repressor factor 1 (BERF1, ZBP-89, and BFCOL1), was first identified in skeletal muscle tissue. Although it represses transcription of the *ENO3* gene by binding to a G-rich box (GTGG(G/C)GGGGGGGTG) in the promoter, it exerts both enhancing and repressing effects on its target genes^11^. The human and mouse *ZNF148* genes have similar promoter sequences and are overall relatively homologous^12^. ZNF148 expression is downregulated during mouse myogenesis and in C2C12 cells^11^. Interestingly, ZNF148 overexpression modestly augments muscle differentiation in C2C12 cells^13^. ZNF148 expression, in concert with reduced Sp1/3, c-Jun, and Stat3 levels, is required for downregulation of vimentin during C2C12 myogenesis^14^. Desmin and vimentin are expressed in regenerating muscle fibers and are the main subunits of fibroblast intermediate filaments. Indeed, they are common to most cells of mesenchymal origin^15^. Vimentin is downregulated during myogenesis, whereas desmin is upregulated as myogenesis progresses. Additionally, ZNF148 indirectly induces expression of cytochrome c oxidase 5b, which is highly expressed in muscle tissue, by regulating the co-interacting partners yin and yang 1 and heterogeneous nuclear ribonucleoprotein d-like protein^16^.

With the goal of identifying positive and negative regulators of muscle differentiation we conducted a screen using a human transcription factor siRNA library, and we identified human ZNF148 as a negative regulator of muscle differentiation *in vitro*. ZNF148 knockdown enhanced and accelerated muscle differentiation in the LHCN-M2 cell line and in primary skeletal muscle myoblasts (HSMM) isolated from human donors. ZNF148 knockdown was sufficient to drive differentiation, even in proliferative conditions. Moreover, *MYOD* and *MEF2C* were rapidly upregulated in these conditions, potentially driving the overall gene program change necessary for myogenesis.

## Results

### An siRNA transcription factor library screen identified ZNF148 as a potential myogenic regulator

**Figure 1:**
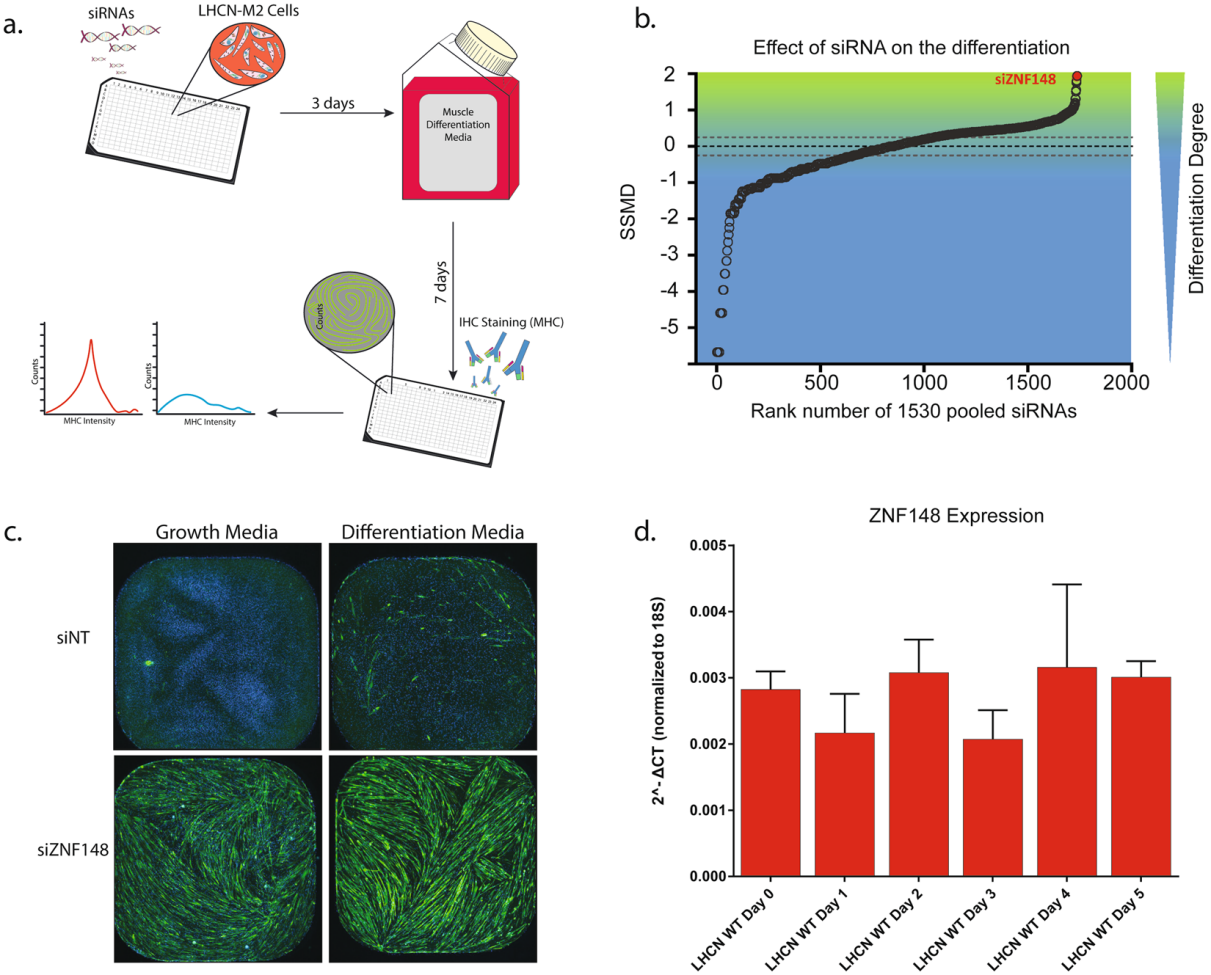
Transcription factor siRNA library screening of LHCN-M2 cells for positive and negative regulators of muscle differentiation. (**a**) Screening design schematic. (**b**) Rank of the strictly standardized mean difference (SSMD) values in the siRNA screen. SSMD values permit statistical scoring of the degree of cell differentiation by considering the area of positive myosin heavy chain (MHC) staining. (**c**) Representative images of cells treated in growth or differentiation culture conditions with nontargeting siRNA (siNT) and the top hit siRNA ZNF148 (siZNF148). (**d**) ZNF148 expression determined by qPCR of LHCN-M2 cells cultivated in either growth (Day 0) or differentiation media (Day 1–5). “WT” represents untreated cells.

To identify repressors and enhancers of muscle differentiation, we conducted a transcription factor library screen, comprising arrayed siRNAs targeting 1530 transcription factors. We introduced siRNAs targeting individual gene (pool of 4 individual siRNAs/gene) into cultures of LHCN-M2 cells, which are nontransformed but are immortalized by telomerase and CDK4 expression^10^ (Fig. 1a). We used a high-content analysis approach by developing an image analysis algorithm to identify and quantify the cell size and number of myosin heavy chain (MHC)-positive cells. To evaluate the effect of the siRNAs on cell differentiation, we calculated strictly standardized mean difference (SSMD) values and ranked them for hit selection (Fig. 1b). The top hit, siZNF148, enhanced MHC expression when the cells were cultivated not only in differentiation media but also in growth media (Fig. 1b,c). *ZNF148* expression remained relatively unchanged during differentiation in nontransfected LHCN-M2 cells (Fig. 1d).

### ZNF148 knockdown efficiency is correlated with enhanced myogenesis

**Figure 2:**
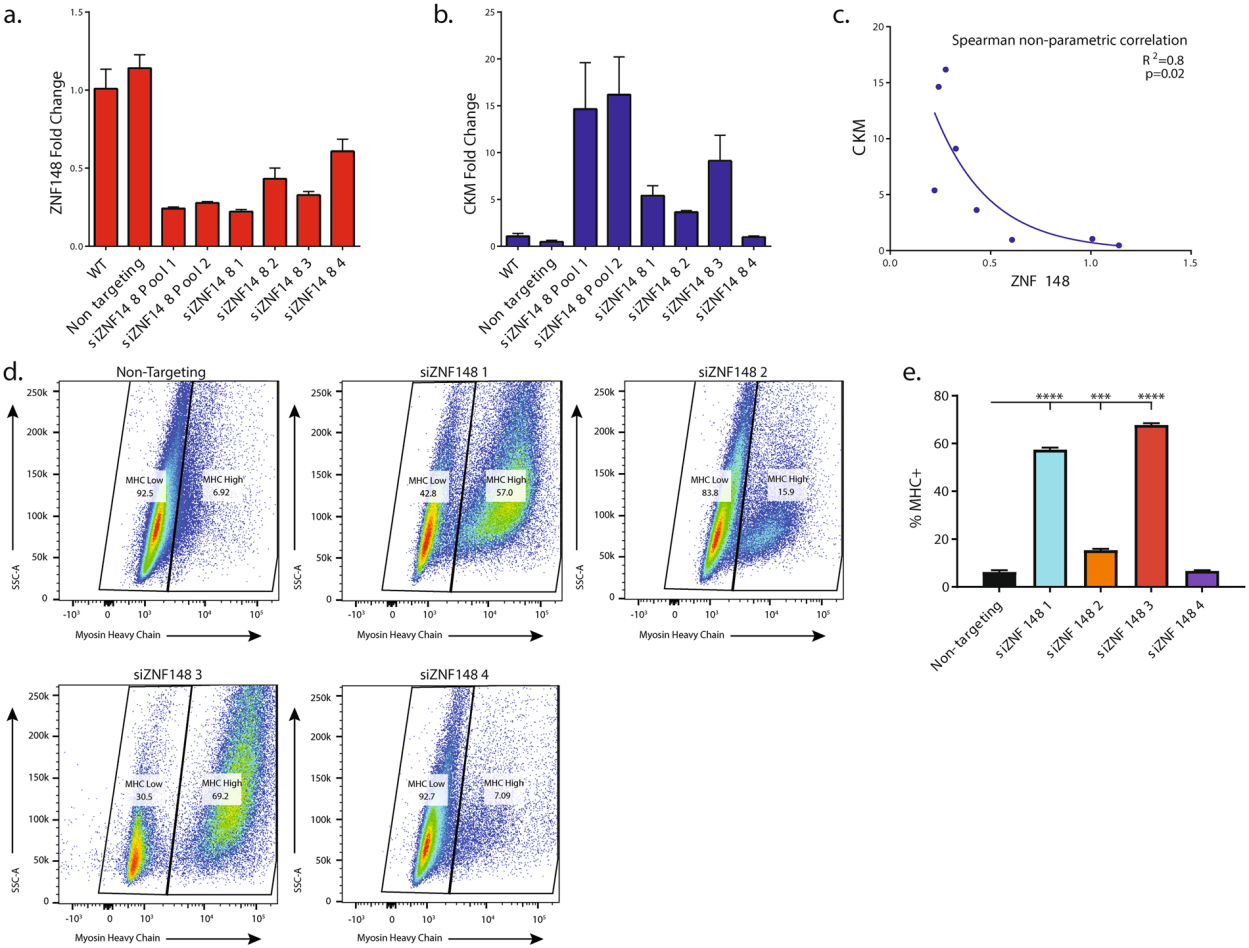
ZNF148 is a negative regulator of human myogenesis. (**a**) *ZNF148* and (**b**) muscle creatine kinase (*CKM*) mRNA expression in LHCN-M2 cells grown for 3 days in differentiation media. Treatment groups include untreated (WT), nontargeting siRNA, two pools containing four siRNAs each, and four individual siRNA sequences. (**c**) Scatterplot and nonlinear fit of *CKM* and *ZNF148* expression levels. Statistics were calculated with a nonparametric Spearman rank-order correlation and a two-tailed *P* value. (**d**) Flow cytometric analysis comparing the level of myosin heavy chain (MHC) expression in LHCN-M2 cells treated with nontargeting or four individual ZNF148 siRNAs. Representative figures are shown, n = 3 independent experiments with 250,000 events collected per experiment. SSC-A stands for side scattered light area. (**e**) Bar graph representing the percentage of cells with high MHC expression levels. Data were analyzed by one-way ANOVA. ***P < 0.01, ****P < 0.001.

Single siRNAs often have off-target effects^17^. To validate that the effect of siZNF148 was not caused by an off-target effect, we investigated the correlation between the levels of ZNF148 and marker of muscle differentiation. We tested the knockdown efficiency of two pools containing four siZNF148s each and four individual siZNF148s by quantitative reverse transcription PCR (qPCR) in LHCN-M2 cells (Fig. 2a). As expected, the individual siRNAs exhibited variable knockdown efficiency, but all siRNAs reduced *ZNF148* expression to some degree. Importantly, we observed a negative association between ZNF148 levels and expression of the muscle differentiation marker muscle creatine kinase (*CKM*) (Fig. 2b,c). By performing a Spearman rank-order correlation nonparametric test, we found that *ZNF148* and *CKM* expression levels were negatively correlated (*r* ^2^ = 0.81, *P* = 0.02). To verify the effect of ZNF148 knockdown on muscle differentiation, we used flow cytometry to analyze MHC protein levels in LHCN-M2 cells after transfecting them with four individual siZNF148s for 2 days and cultivating them in differentiation (i.e., growth factor–depleted) media for an additional 2 days (Fig. 2d,e and Supplementary Fig. S1). Three of the four individual siRNAs enhanced LHCN-M2 differentiation (Fig. 2e). The morphology of the differentiated cells (Supplementary Fig. S1) was concordant with the expression levels of *CKM* mRNA and MHC protein (Fig. 2). Contrary to previous observations in the mouse C2C12 cell line^13^, *ZNF148* overexpression did not alter myogenesis in LHCN-M2 cells (Supplementary Fig. S1). It is unclear whether this difference is caused by differential endogenous levels of ZNF148 protein in the two cell lines or if it is a reflection of the complex and dynamic biology of transcription factor regulation during myogenesis.

### Downregulation of ZNF148 induces muscle differentiation in immortalized human myoblasts and primary human skeletal myoblasts

**Figure 3:**
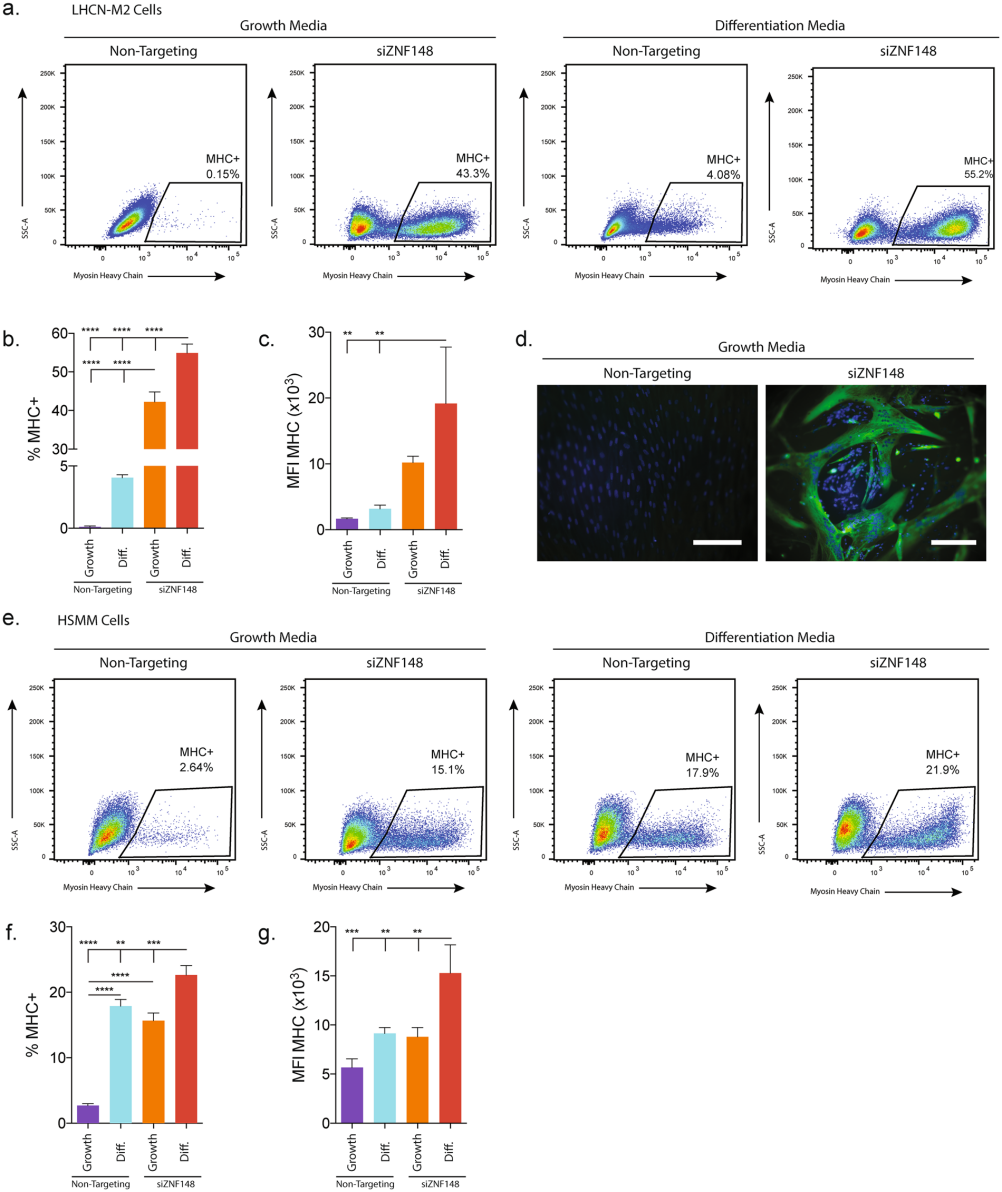
ZNF148 knockdown differentiates cells in proliferative media and is not cell type specific. (**a**) Flow cytometric analysis of myosin heavy chain (MHC) expression in LHCN-M2 cells cultivated for 2 days in growth or differentiation media. (**b**) Quantification of cell population percentages or (**c**) mean fluorescence intensity (MFI) of MHC-high expressing LHCN-M2 cells. Statistical significance was determined by one-way ANOVA. (**d**) Fluorescence microscopy of MHC- (green) and DAPI- (blue) stained LHCN-M2 cells after culture for 3 days in growth media. Scale bar = 50 µM. (**e**) Flow cytometric analysis of MHC expression in primary human muscle myoblasts (HSMM) cells cultivated for 4 days in growth or differentiation media. (**f**) Quantification of cell population percentage and (**g**) mean fluorescence intensity (MFI) of MHC-high expressing LHCN-M2 cells. Representative figures are shown, n = 3 independent experiments with 250,000 events collected per experiment. Data were analyzed by one-way ANOVA. **P < 0.01, ***P < 0.001, ****P < 0.0001. SSC-A stands for side scattered light area.

ZNF148 knockdown induced robust differentiation even under nondifferentiation culture conditions (Fig. 1c). To confirm that ZNF148 inhibits muscle differentiation, we used flow cytometric analysis to quantify the levels of MHC on a per cell basis (Fig. 3). Transfection of LHCN-M2 cells with siZNF148 quickly induced differentiation in growth media, whereas nontargeting siRNA had no effect (Fig. 3a). Although this effect was further enhanced by differentiation media, siZNF148 was the major driver of differentiation in both growth and differentiation media, as cultivating LHCN-M2 cells transfected with nontargeting siRNA in differentiation media produced a smaller population of differentiated cells than that of siZNF148 (Fig. 3a–c). Transfection of LHCN-M2 cells with siZNF148 also induced formation of MHC-positive filaments in multinucleated myotube structures (Fig. 3d).

We next confirmed the effect of ZNF148 knockdown in primary HSMM cells. Consistent with our findings in LHCN-M2 cells, siZNF148 robustly induced high levels of MHC protein expression, indicative of HSMM differentiation (Fig. 3e–g). The gating strategy used to identify the expression of MHC in LHCN-M2 and HSMM by flow cytometry is illustrated in Supplementary Fig. S2. In both LHCN-M2 and HSMM cells, siZNF148 also induced high levels of myogenin by using qPCR (Fig. S2c–f).

### ZNF148 knockdown activates master myogenic transcription factors to induce myogenesis

**Figure 4:**
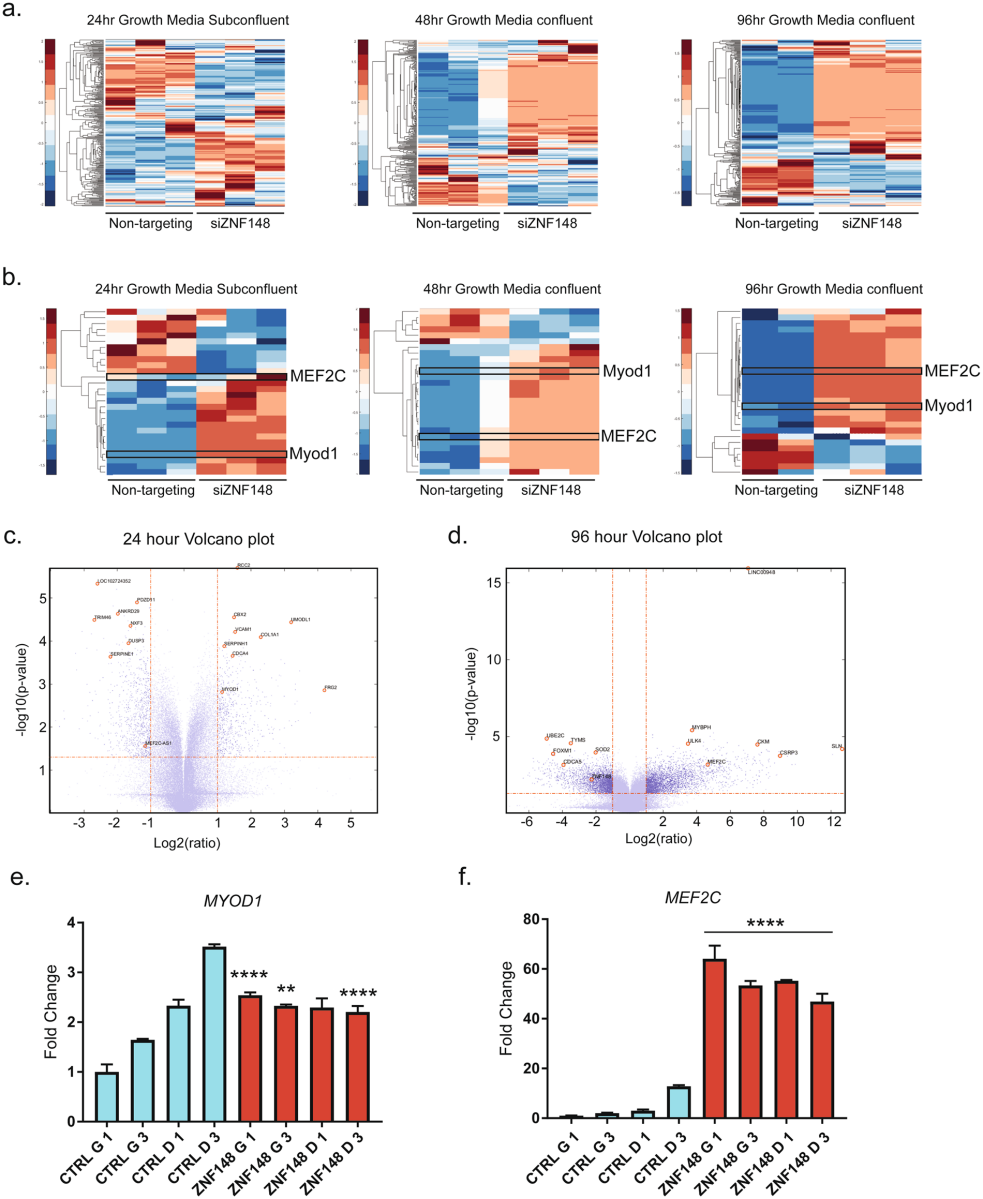
Microarray analysis reveals prominent muscle transcription factors are upregulated within 24 hours of ZNF148 knockdown. (**a**). Microarray clustergram of 269 muscle genes in LHCN-M2 cells cultivated for 24 hours (subconfluent), 48 hours (confluent), or 96 hours (confluent) in growth media. Nontargeting siRNA was compared with ZNF148 siRNA. (**b**) Microarray clustergram of the statistically significant (*P* < 0.05) muscle genes in LHCN-M2 cells cultivated for 24 hours in growth media (subconfluent). The same genes are shown for cells cultivated for 48 hours (confluent) and 96 hours (confluent) in growth media. Nontargeting siRNA was compared with ZNF148 siRNA. Volcano plots of LHCN-M2 cells grown for (**c**) 24 hours and (**d**) 96 hours. The plots illustrate all of the top hits from the microarray analysis at indicated times. (**e**) *MYOD* expression in LHCN-M2 cells transfected with either nontargeting siRNA or ZNF148 siRNA in growth media (G) or differentiation media (D) for 1 or 3 days (G1, G3, D1 and D3). (**f**) *MEF2C* expression in LHCN-M2 cells treated with the conditions described in (**e**). **P < 0.01, ***P < 0.001, ****P < 0.0001.

To determine the early driver genes responsible for the siZNF148-induced myogenic phenotype, we performed DNA microarray analysis of LHCN-M2 cells transfected with siZNF148 at different time points. We examined the expression levels of 269 muscle-related genes in response to siZNF148. Gene set enrichment and gene ontology analyses revealed a strikingly similar upregulation pattern that was time dependent (Supplementary Fig. S3). To determine the differential expression of the genes after 24, 48, and 96 hours of ZNF148 knockdown, we generated histograms of respective *t* scores and *P* values, allowing estimation of the *t*-value distribution (Supplementary Fig. S4). A subset of these genes was robustly upregulated after 48 and 96 hours, but not after 24 hours (Fig. 4a). *MEF2C* and *MYOD* were significantly upregulated (*P* > 0.05) at each time point after ZNF148 knockdown (Fig. 4b).

We observed that a disproportionate number of upregulated genes clustered to the chromosome location 6p22 after 24 hours of ZNF148 knockdown (Supplementary Fig. 4). Interestingly, genes that were downregulated after 48 and 96 hours clustered to the same chromosome location with considerable gene overlap. The genes in this cluster were largely histone genes (Supplementary Table S1), which may play a role in initiating the myogenic program. To better visualize the expression difference between the 24- and 96-hour time points, we generated volcano plots (Fig. 4c,d), which revealed several other genes that are drastically up or downregulated. One such example is an important regulator of skeletal muscle physiology termed *MRLN* ^18^ (Supplementary Data Set).

To validate our microarray findings, we conducted qPCR analysis of nontargeting and ZNF148 siRNA–transfected LHCN-M2 cells cultivated in either growth or differentiation media. As expected, *MYOD* was upregulated (2.5×) within 24 hours of ZNF148 knockdown and remained at approximately the same level, regardless of growth or differentiation conditions. In contrast, *MYOD* levels steadily increased in nontargeting siRNA–treated cells grown in differentiation media (Fig. 4e). Furthermore, *MEF2C* was greatly upregulated (~60×) within 24 hours of ZNF148 knockdown (Fig. 4f). These findings illustrate that *MEF2C* and *MYOD* play a critical role in muscle differentiation. This is particularly supported by evidence that ectopic expression of *MYOD* in nonmuscle cell types results in a muscle phenotype^4, 19, 20^ and that dysregulated *MYOD* is primarily responsible for impaired differentiation in rhabdomyosarcoma^21^.

## Discussion

The overall objective of these studies was to identify the regulators of human muscle differentiation. To meet this objective, we used human LHCN-M2 cells rather than mouse C2C12 cells, which eliminated the identification of nonconserved regulators. We found the most robust negative regulator of muscle differentiation was ZNF148. Similar observation was made in HSMM cells, although the effect of siZNF148 in HSMM was slightly weaker than that in LHCN-M2 cells. LHCN-M2 cells were derived from a selected clone that displayed a tremendous ability to differentiate^10^, which may explain the more striking effect of ZNF148 knockdown in LHCN-M2 cells. Nevertheless, knockdown of ZNF148 results in a strong enhancement of differentiation and is not cell line specific.

ZNF148 regulates differentiation of several other tissue types^22–24^ in addition to muscle^11, 13^. However, the role of ZNF148 in muscle differentiation may be species specific. Although downregulation of ZNF148 in mouse C2C12 delays myogenesis by inhibiting the required downregulation of vimentin, Sp1, and, Sp3^13, 14^, we show that the opposite phenomenon occurs during human myogenesis. Because ZNF148 is highly conserved, it is likely that the differences between human and mouse are mediated by diverse downstream targets of ZNF148 or by splice variants of full-length ZNF148^12^. Additionally, the level and tissue distribution of ZNF148 expression may be different among species. For example, mice express low levels in the testis and heart, intermediate levels in skeletal muscle, and high levels in the brain and liver^11^. Moreover, ZNF148 levels are downregulated in mouse embryos and in C2C12 cells. In contrast, rats express similar levels of ZNF148 among all tissues^23^. Likewise, ZNF148 levels remain relatively unchanged during human LHCN-M2 cell differentiation.

It is important to note that overexpression of *ZNF148* did not affect MHC expression in LHCN-M2 cells, unlike that in C2C12 cells^13^. We observed cellular stress and growth defects in response to *ZNF148* overexpression, which ultimately led to cell death (data not shown). However, when we transduced LHCN-M2 cells with diluted lentiviral-ZNF148 cDNA, the cells were able to grow and differentiate; suggesting that constitutive expression of high levels of ZNF148 protein is cytotoxic and impairs myogenesis. In addition, ZNF148 levels are dynamic in embryo development, in which they are upregulated during early embryogenesis and rapidly downregulated in fetal development^11^. It is likely that C2C12 and LHCN-M2 cells do not recapitulate identical stages of myoblast differentiation. Therefore, ZNF148 may play different roles at different stages of myoblast to myotube progression.

We used DNA microarray analysis at different time points after ZNF148 knockdown to identify genes that are upregulated during myogenesis in a time-dependent manner. We identified an early downregulation response followed by a late upregulation response of genes involved in muscle differentiation. The two most notable early response genes were *MYOD* and *MEF2C*. Together, our findings suggest that ZNF148 knockdown induces changes in the transcriptome that lead to direct or indirect upregulation of *MYOD* and *MEF2C*, resulting in commitment to a muscle differentiation program. However, further studies are required to elucidate the exact mechanism in which ZNF148 regulates MYOD/MEF2C function.

Here, we present evidence that ZNF148 knockdown induces global gene expression changes, which result in an enhanced myogenic program. Remarkably, we observed a specific early- and late-response change in the expression of histone genes. Although it is presumable that these genes play some role in the initiation of myogenesis, to our knowledge histone genes have not been studied during muscle differentiation, nor have they been implicated as interacting partners of ZNF148. Because these genes appear to be specifically regulated during myogenesis, possibly in a temporal manner, the role these genes play in muscle determination and differentiation warrants further investigation.

ZNF148 plays a significant role in differentiation, likely through the maintenance of a pluripotent state by inhibiting differentiation proteins MYOD and MEF2C. Thus, ZNF148 regulation may allow for normal growth and repair of muscle cells. ZNF148 has been described as both a positive and negative regulator and although ZNF148 transcriptional level does not change, downstream targets likely change, which has been shown previously^16^. Aberrant or unregulated ZNF148 expression may contribute to diseases of muscle differentiation, such as rhabdomyosarcoma.

## Methods

### Materials

Fetal bovine serum and horse serum were purchased from HyClone (Logan, UT). Cell culture reagents, fluorescent secondary antibodies, Lipofectamine, and RNAiMax were purchased from Invitrogen (Carlsbad, CA). Zinc sulfate, Vitamin B12, apo-transferrin, dexamethasone, and insulin were purchased from Sigma-Aldrich (St. Louis, MO). Hepatocyte growth factor (GF116) was purchased from EMD Millipore (Billerica, MA). Bovine fibroblast growth factor was purchased from BioPioneer Inc. (HRP-0011). Pooled (L-012658-00-005), individual ZNF148 (D-012658-01, D-012658-02, D-012658-04, D-012658-17), and nontargeting siRNAs (D-001210-05-05) were purchased from GE Dharmacon (Lafayette, CO). Alexa Fluor 700 mouse IgG2a isotype control, Alexa Fluor 647 mouse IgG1isotype control, Alexa Fluor 488 mouse IgG1isotype control, PE mouse IgG2b isotype control, PE-MHC, BD Phosflow, and BD Cytofix were purchased from BD Biosciences (San Jose, CA). Human MHC antiserum was collected from an MF20 hybridoma (Developmental Studies Hybridoma Bank, Iowa City, IA). MYH2 (MHC), ZNF148, CKM, MYOG, GAPDH, and 18S TaqMan probes were purchased from Thermo Fisher Scientific (Waltham, MA).

### Cell culture

All cell lines were maintained in a humidified incubator at 37 °C with 5% CO_2_. LHCN-M2 cells were grown and differentiated as previously described^10^. HSMM cells were purchased from Lonza (Allendale, NJ) and cultivated according to manufacturer protocol. The cells were routinely verified to be mycoplasma-free by using the MycoProbe Mycoplasma Detection kit (R&D Systems, Minneapolis, MN).

### siRNA screen

The general procedure for siRNA screen and analysis has been previously described^25^. The human transcription factor library siRNAs (GE Dharmacon) were reverse-transfected into LHCN-M2 cells with DHARMAFect2 (GE Dharmacon) at a final concentration of 25 nM in gelatin-coated 384-well plates (Perkin Elmer, Waltham, MA). At 24 hours after transfection, the cells were supplemented with complete growth media (a four to one ratio of DMEM to M199 supplemented with 15% FBS, 0.02 M HEPES, 0.03 µg/mL zinc sulfate, 1.4 µg/mL vitamin B12, 0.055 µg/mL dexamethasone, 2.5 ng/ml hepatocyte growth factor, and 10 ng/ml basic-FGF). To initiate the differentiation of myoblasts, one-half of the growth media was substituted with differentiation media (a four to one ratio of DMEM: to M199 supplemented with 0.02 M HEPES, 0.03 µg/mL zinc sulfate, 1.4 µg/mL vitamin B12, 10 µg/mL insulin, and 100 µg/mL apo-transferrin). One-half of the media was exchanged every 48 hours. After 7 days, the cells were fixed in 4% paraformaldehyde (EMS, Hatfield, PA), permeabilized with 0.25% Triton X-100 in PBS, and incubated with MHC antiserum and anti–FLAG M2 antibody overnight at 4 °C. After a 1-hour incubation with a goat anti–mouse secondary antibody conjugated with Alexa Fluor 488 and with DAPI, the cells were imaged with the Acumen Cellista system (TTP Labtech, Cambridge, MA). Intensity, area and count of nuclei, and MHC-positive cells were measured with the Cellista program. An SSMD-based algorithm on a graphical user interface, termed GUItars^26^, for analysis of high-throughput RNA interference screening was used for statistically evaluating quality controls and effects of the test siRNAs. Hits were identified as putative enhancers (SSMD ≤ −0.5).

### RNA extraction and quantitative reverse transcription PCR

RNA was extracted with a Maxwell simplyRNA Tissue kit and Maxwell Instrument (Promega, Madison, WI). RNA concentration was measured with a NanoDrop 8000 UV-Vis Spectrophotometer (Thermo Fisher Scientific). The SuperScript VILO cDNA Synthesis kit (Life Technologies, Carlsbad, CA) was used to synthesize cDNA, according to the manufacturer protocol. To determine mRNA expression, Applied Biosystems TaqMan assays (20×), Fast Advanced Master Mix (Life Technologies), and an Applied Biosystems 7900HT Fast Real-Time PCR system (Life Technologies) were used in accordance with the TaqMan Fast protocol. Gene expression was normalized to the 18S rRNA housekeeping gene, which did not vary in expression during LHCN-M2 or HSMM cell differentiation. Each experiment was performed at least three times, and all samples were analyzed in triplicate.

### siRNA transfection (6-well plates)

Cells were transfected with 20 nM siRNA per well with RNAiMax (Invitrogen), according to the manufacturer protocol. In differentiation experiments, the media was replaced with differentiation media 2 days later, effectively removing the transfection reagents. For 24-hour microarray samples, the transfection reagents were removed during media washes prior to RNA extraction.

### Lentivirus generation and viral transduction

Lentivirus was generated in HEK293T cells (ATCC, Manassas, VA) in 10-cm dishes. Briefly, 9 µg human ZNF148 transfer vector (GE Dharmacon), 7 µg psPax2 (plasmid # 12260, Addgene, Cambridge, MA), and 2 µg pmd2.G (plasmid # 12259, Addgene) were combined with lipofectamine 3000, according to the manufacturer protocol. Virus was collected 48 hours after transfection, centrifuged at 500 *g* for 5 min to remove cells and debris, filtered with a 0.45 µM PES filter, and frozen at −80 C. Viral transduction was accomplished with 500 μL viral media added to one 10 cm-dish of LHCN-M2 cells at 75% cellular confluence with 4 µg/mL Polybrene (Sigma-Aldrich) for 16 hours. Viral media was then replaced with fresh growth media.

### Flow cytometry

To determine the degree of muscle differentiation, we performed flow cytometric analysis of MHC staining. LHCN-M2 (10^6^) and HSMM cells (10^6^) were fixed with BD Cytofix and permeabilized with BD Phosflow, according to manufacturer directions. The cells were washed three times with 1 mL staining buffer (1 × DPBS, 2% FBS, 1 mM EDTA, and 0.02% sodium azide) and then stained with PE-MHC (1 ng/10^6^ cells) and DAPI for 20 min at room temperature while protected from light. After staining, the cells were washed twice with staining buffer, resuspended in 500 µL staining buffer and analyzed with a custom-configured BD Fortessa flow cytometer and FACSDiva software (BD Biosciences). Data were analyzed with FlowJo software v10 (TreeStar, Ashland, OR). All experiments were performed with at least three biological replicates and 250,000 events collected per sample.

### Immunofluorescence and microscopy

LHCN-M2 cells were grown and differentiated in gelatin-coated chamber slides. The cells were fixed with 4% paraformaldehyde and then permeabilized with 0.1% Triton × 100 for 10 to 15 min. The cells were blocked with 3% BSA for 1 hour at room temperature and then stained with MHC primary antibody overnight. The cells were washed three times with TBST buffer (50 mM Tris, 150 mM NaCl, 0.1% Tween 20). After washing, the cells were stained with a secondary antibody conjugated with Alexa Fluor 488 for 1 hour at room temperature. After staining, the cells were washed three times with TBST and then stained with DAPI (300 nM) for 3 min, followed by three more washes with TBST. The stained cells were mounted with Prolong Gold antifade reagent (Life Technologies). Images were acquired with a Nikon Eclipse Ti-S fluorescence microscope (Nikon Instruments Inc., Melville, NY).

### Microarray

The general DNA microarray procedure has been described previously^27^. LHCN-M2 cells were transfected with 20 nM ZNF148 siRNA or nontargeting siRNA. After 24 hours of transfection, cell pellets were frozen at −80 C. Additional cell pellets were frozen after 48 or 96 hours in growth media or differentiation media. The pellets were lysed and RNA was extracted with the Qiagen miRNAeasy kit, according to the manufacturer protocol. The RNA was further cleaned with the Qiagen RNeasy MinElute kit, according to the manufacturer directions. RNAs were amplified and labeled with the One-Color Low Input Quick Amp Labeling kit (Agilent 5190–2305, Santa Clara, CA) followed by hybridizing to the SurePrint G3 Human GE 8 × 60 K microarray (Agilent-028004) that contains 42,545 unique probes targeting 27,958 Entrez genes. Microarrays were scanned with an Agilent array scanner (G2565CA) at 3-μm resolution. Microarray data were extracted by Agilent Feature Extraction software (v10.5.1.1) and the GE1_107_Sep09 protocol. Quantile normalization on log-transformed, background-subtracted signal intensity was performed for all samples, followed by comparison between the sets of replicates from different experimental groups. Student *t* test was used to determine the statistical significance of the difference between the paired samples from three replicates of each experiment. The expression of a gene was considered significantly different if the *P* value was less than 0.05 and the expression change was greater than two-fold for at least one of the group comparisons. The data process and PCA analysis were performed using Partek software (St. Louis, MO). Geo no. GSE94441.

### Heatmaps, volcano plots, gene set enrichment analysis, and gene ontology

We used the clustergram syntax in MATHLAB software to compute hierarchical clustering representations of select genes as a function of relative expression. Color bars were inserted to show the scale of comparative expression values. We conducted word searches within known gene functions and identified 269 known muscle-related genes. Volcano plots were generated in MATLAB by plotting statistically significant differentially expressed genes for each group. Statistically significant genes were plotted as a function of −log10 (*P* value), and significant differentially expressed genes were plotted by log2. Significant differences in expression were identified as values occurring outside the fold change of log2(ratio) values. Significantly upregulated or downregulated gene lists from each group were subjected to enrichment analysis using the Enrichr web server (http://amp.pharm.mssm.edu/Enrichr)^28, 29^. The ‘network’ and ‘chromosome location’ features were utilized to generate gene set networks and chromosomal loci representations, respectively. ‘GO Biological Process’ was chosen for all shown networks. Graphical scatterplot representations were generated by placing enriched gene sets into the REVIGO web server^30^ to visualize nonredundant, significantly upregulated genes for all time points. Color, size, and both axes correspond to log10 *P* value.

### Statistical analysis

Datasets obtained from DNA microarray analysis were subjected to two-sample *t* tests with MATLAB software. Values were subjected to 10,000 permutations and re-evaluated with *t* tests to examine differential expression of genes from the 24-, 48-, and 96-hour time points. Histograms of respective *t* scores and *P* values were generated to allow estimation of the *t*-value distribution. Statistically significant genes were selected in each group when *P*  < 0.05. To ascertain whether a correlation exists between *CKM* and *ZNF148* expression levels, we created a scatterplot and used a nonlinear line to visualize the fit. We then performed a Spearman rank-order correlation nonparametric test to generate a correlation coefficient. For other assays, data from at least three independent replicated experiments were quantitatively analyzed by one-way ANOVA with Dunnett multiple comparisons test or by Student 2-tailed *t* test with GraphPad Prism 7.0 software, as indicated. All data are expressed as the mean ± SD.

### Data availability

The authors declare that all data supporting the findings of this study are available within the article and its Supplementary Information, or from the corresponding author on request. The accession code is: Geo no. GSE94441 for Microarray analysis (GEO accession number).

## Electronic supplementary material

Supplementary Information

Supplementary Dataset 1
