# Supplementary material for: Transcription factor ZNF148 is a negative regulator of human muscle differentiation
Source: Sci Rep. 2017 Aug 15;7:8138. doi: 10.1038/s41598-017-08267-5 (PMC5557752; doi:10.1038/s41598-017-08267-5)
Supplement: Supplementary file 1 — Supplementary Information [file 41598_2017_8267_MOESM1_ESM.pdf]

Transcription factor ZNF148 is a negative regulator of human muscle differentiation

Jesse Bakke[1], William C. Wright[1,2], Anthony E. Zamora[3], Su Sien Ong[1], Yue-Ming Wang[1], Jessica D. Hoyer[1,2], Christopher T. Brewer[1,2], Paul G. Thomas[3] & Taosheng Chen[1,2,*]

[1]Department of Chemical Biology and Therapeutics, St. Jude Children's Research Hospital, Memphis, Tennessee, USA.

[2]Integrated Biomedical Sciences Program, University of Tennessee Health Science Center, Memphis, Tennessee, USA.

[3]Department of Immunology, St. Jude Children's Research Hospital, Memphis, Tennessee, USA.

*Correspondence and requests for materials should be addressed to T. C. (email: taosheng.chen@stjude.org)

Supplementary Figure S1. (**a**) Phase-contrast microscopy of LHCN-M2 cells after culture for 3 days in differentiation media. Treatment groups include untreated (WT), nontargeting siRNA, two pools containing four siRNAs each, and four individual siRNA sequences. (**b**) Representative gating strategy for flow cytometric analysis of myosin heavy chain (MHC) staining, as described in Fig. 2d. (**c**) *ZNF148* expression and (**d**) *MHC* expression in LHCN-M2 cells treated with the following constructs: GFP, ZNF148 cDNA (oeZNF148), nontargeting siRNA (siControl), and ZNF148 siRNA. Data was analyzed by one-way ANOVA. ****$P < 0.0001$.

Supplementary Figure S2. Representative gating strategy for flow cytometric analysis of myosin heavy chain (MHC) staining in (**a**) LHCN-M2 cells and (**b**) primary human muscle myoblasts (HSMM), as described in Fig. 3a,e. (**c**) *ZNF148* expression and (**d**) *MYOG* expression in LHCN-M2 cells treated with nontargeting siRNA (siControl), and ZNF148 siRNA. (**e**) *ZNF148* expression and (**f**) *MYOG* expression in HSMM cells treated with nontargeting siRNA (siControl), and ZNF148 siRNA. Data was analyzed by one-way ANOVA. ****$P < 0.0001$.

Supplementary Figure S3. Graphical scatterplots and ontology networks of enriched significantly upregulated genes from (**a**) 24-hour, (**b**) 48 hour, and (**c**) 96-hour time points. Size, color, and both axes correspond to the log 10 *P* values of upregulated genes in scatterplots.

Supplementary Figure S4. (**a**) Histograms of *t*-score and *P*-value distributions following 10,000 permutations of expression values. (**b**) Visualization of genes mapped to chromosome location for enriched statistically significant genes that were upregulated at 24 hours (red), downregulated

at 48 hours (orange), and downregulated at 96 hours (blue).

Supplementary Table S1. Complete list of upregulated (24 hour) and downregulated (48 and 96 hour) genes located on chromosome 6p22.

Supplementary Data Set. Complete list of differentially expressed genes in microarrays from LHCN-M2 cells grown for 24, 48, and 96 hours after ZNF148 knockdown. Raw data are included.

Supplementary Figure S1

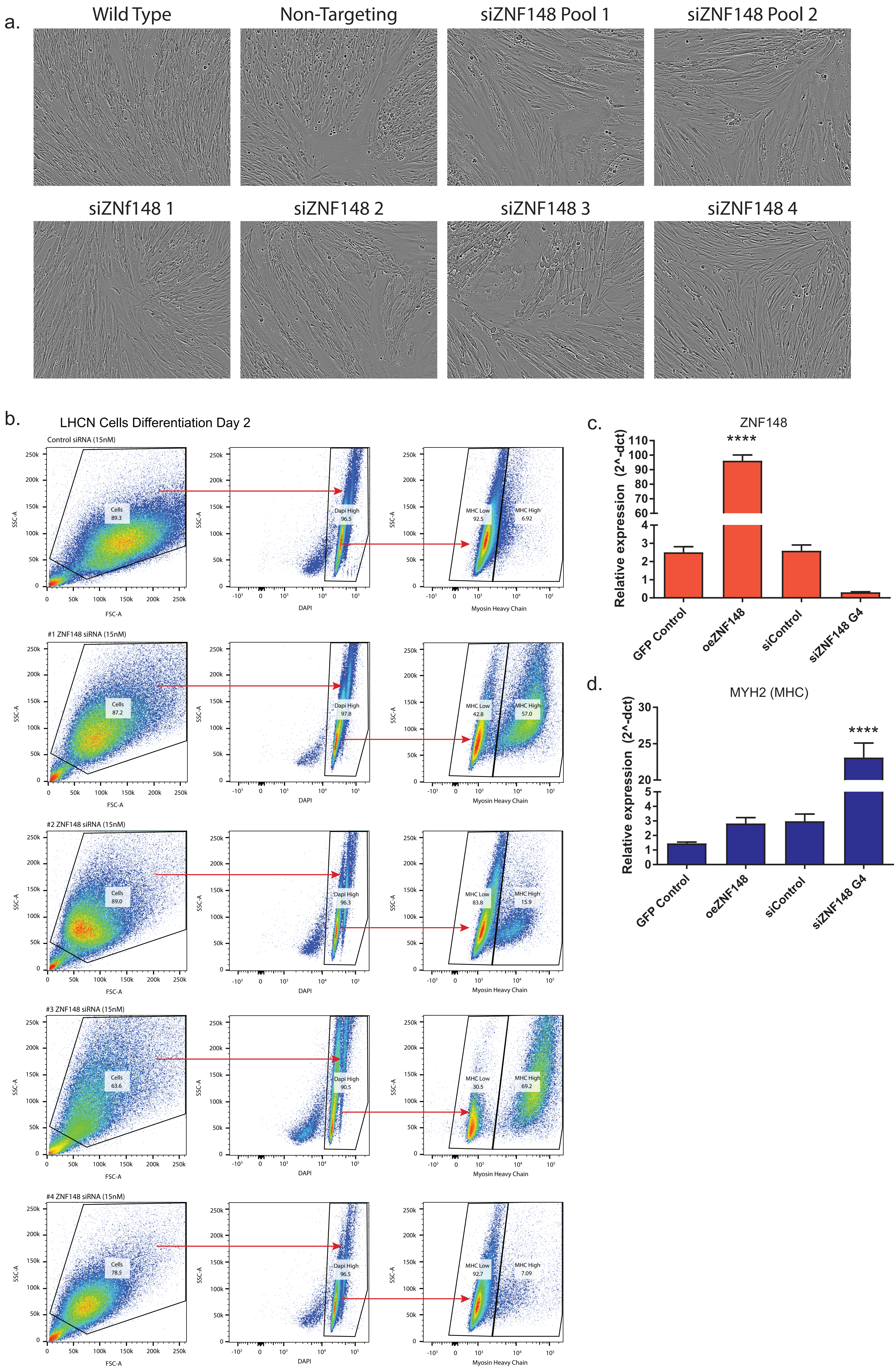

Supplementary Figure S2

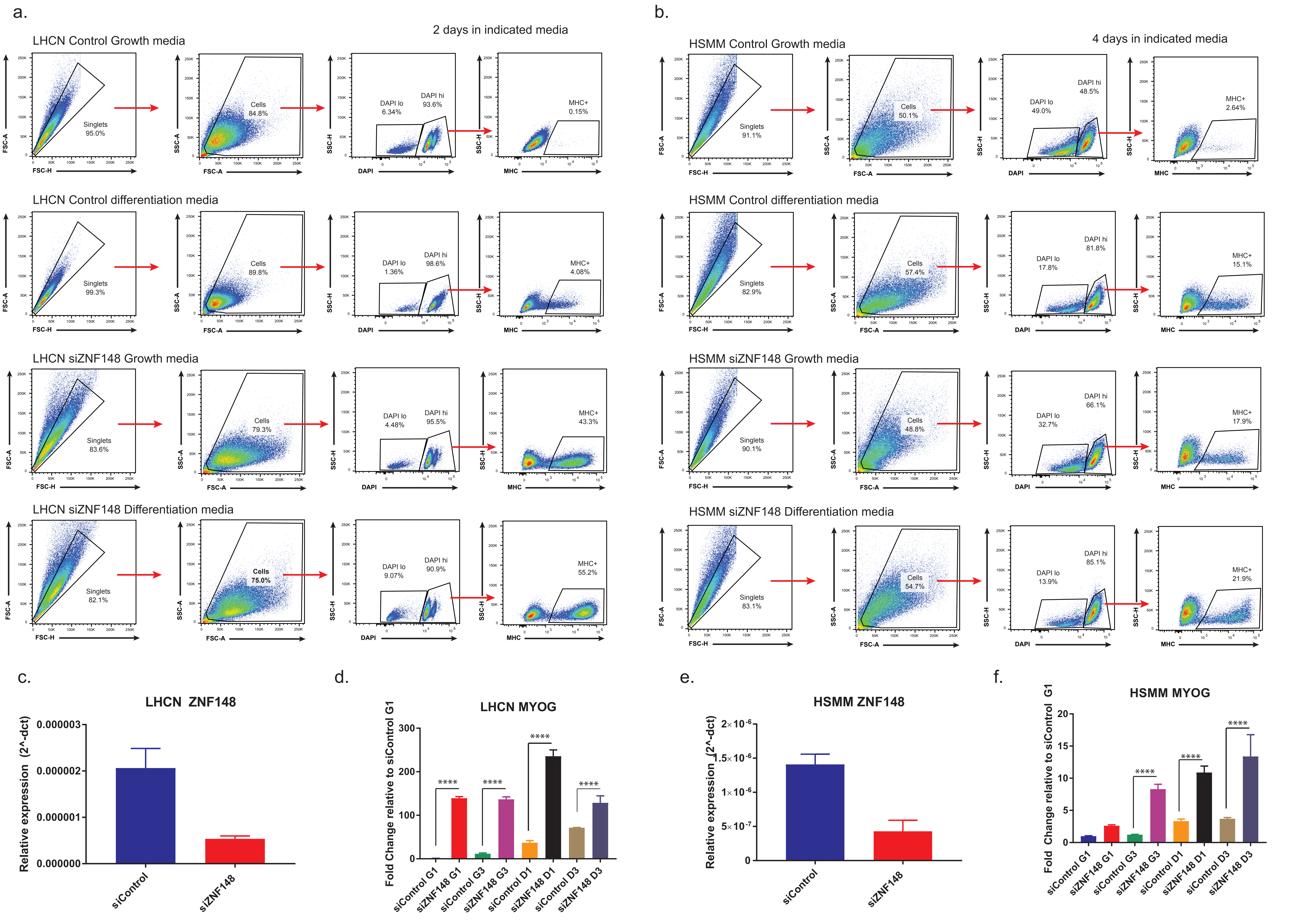

Supplementary Figure S3

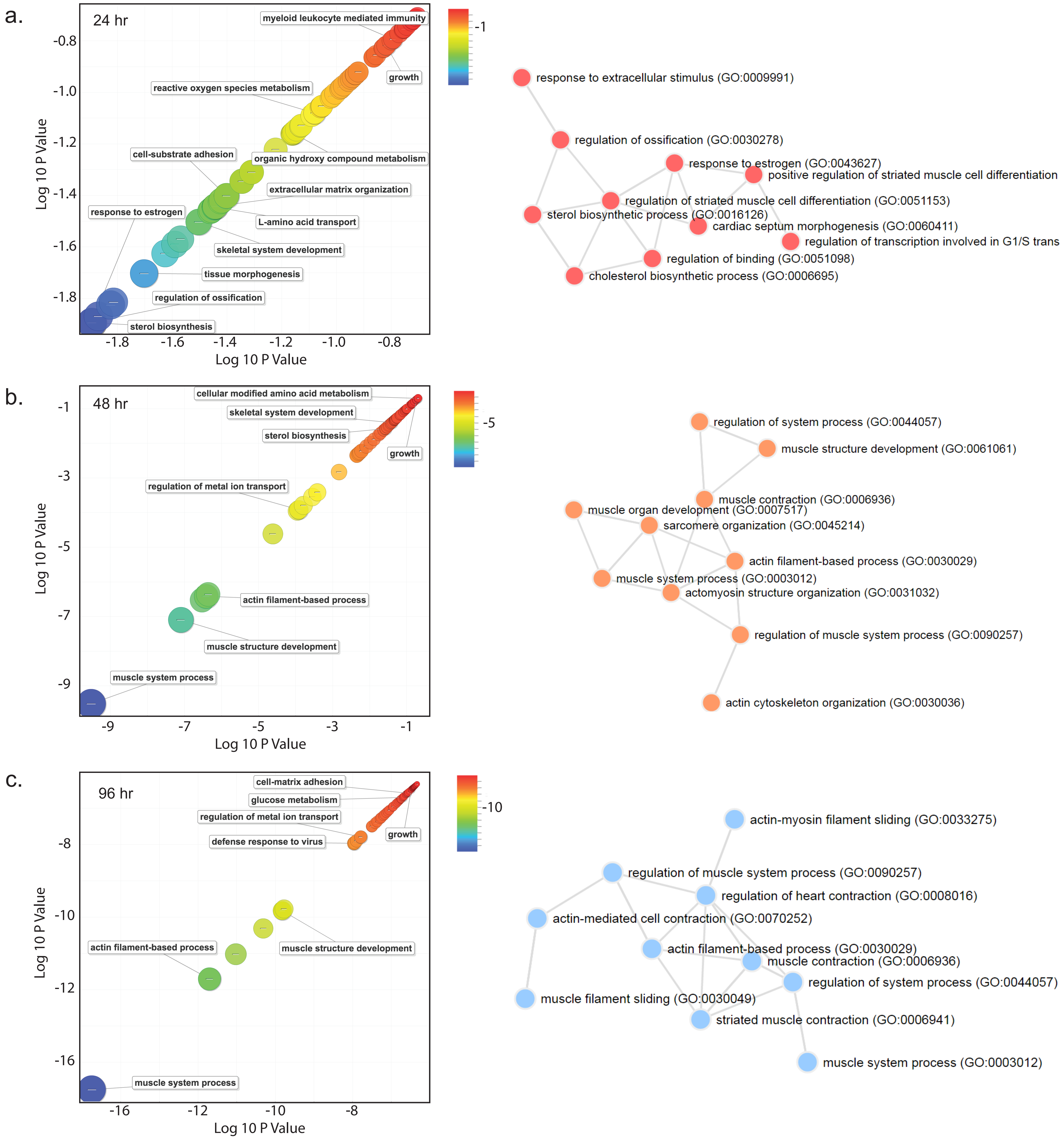

Supplementary Figure S4

a.

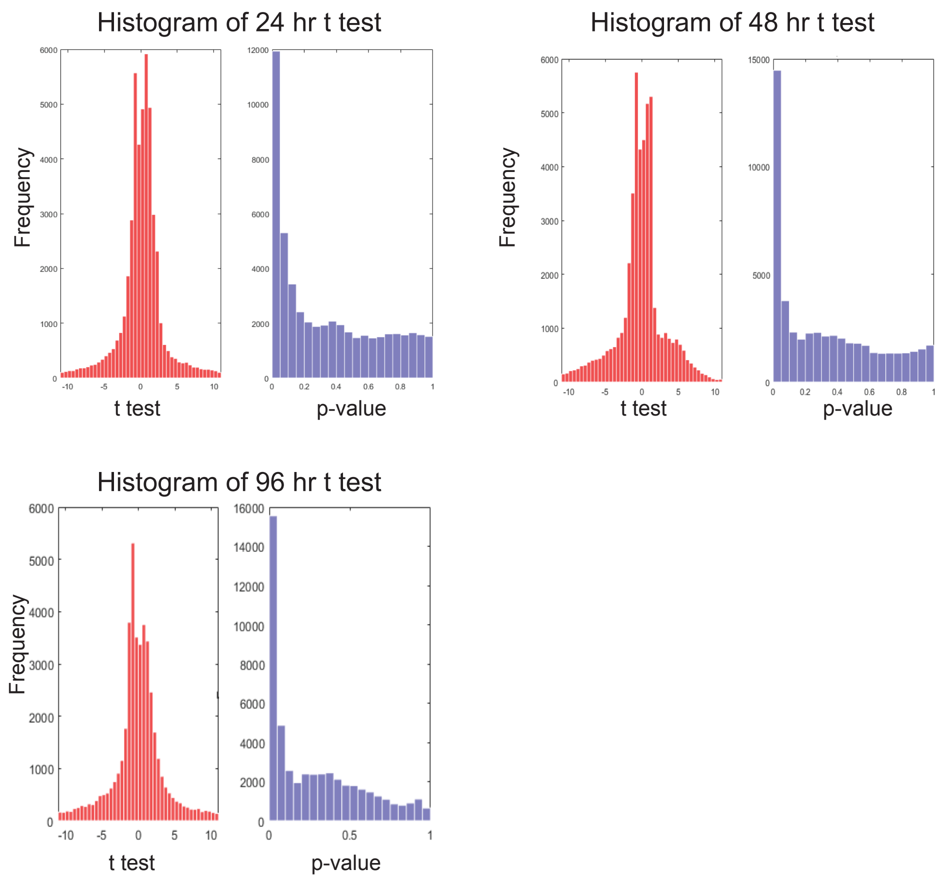

b.

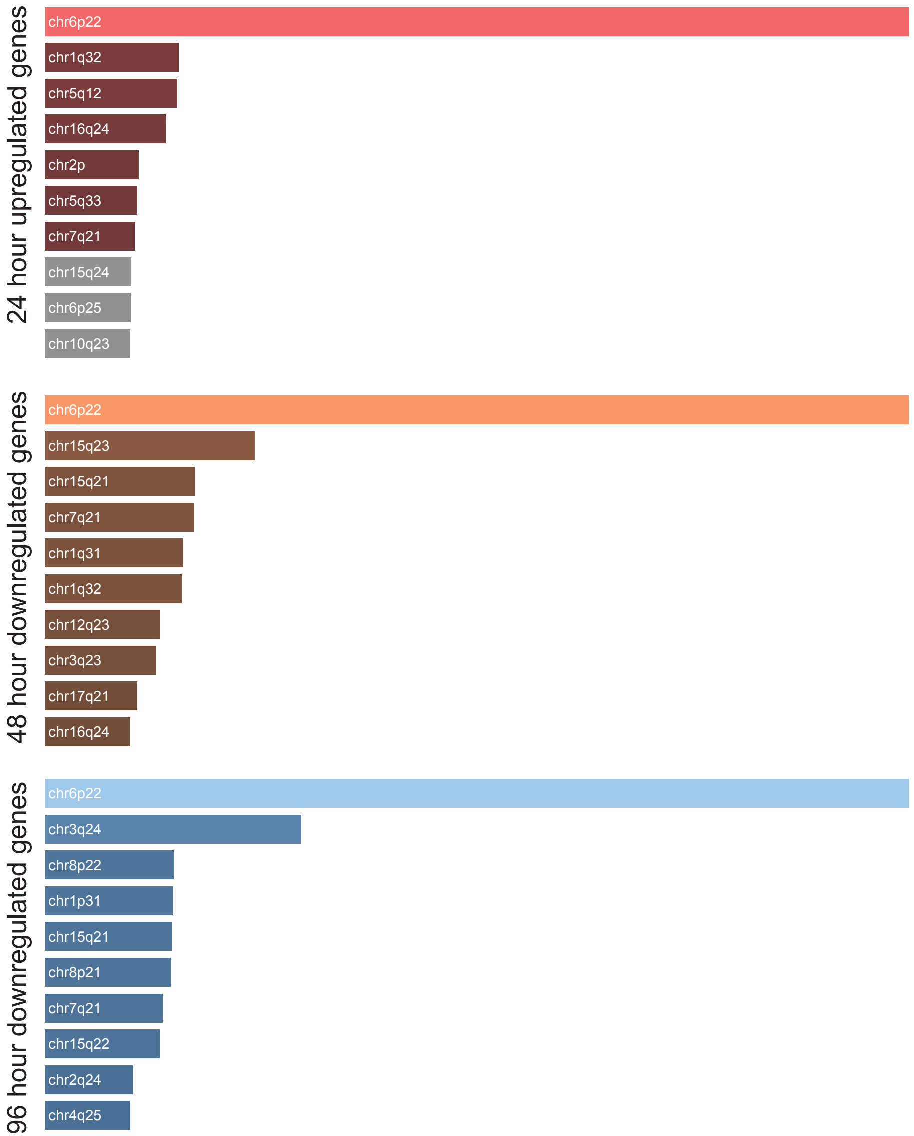

Supplementary Table S1.

24hr 6p22 upregulated genes	48hr 6p22 downregulated genes	96hr 6p22 downregulated genes
HIST1H1A	ATXN	BTN2A2
HIST1H1B	BTN2A2	HIST1H1A
HIST1H2AB	BTN3A1	HIST1H1B
HIST1H2AG	BTN3A2	HIST1H1D
HIST1H2AI	CAP2	HIST1H2AB
HIST1H2AL	HIST1H1A	HIST1H2AG
HIST1H2BE	HIST1H1B	HIST1H2AH
HIST1H3B	HIST1H1D	HIST1H2AI
HIST1H3D	HIST1H2AB	HIST1H2AJ
HIST1H3F	HIST1H2AC	HIST1H2AK
HIST1H3H	HIST1H2AD	HIST1H2AL
HIST1H3I	HIST1H2AE	HIST1H2AM
HIST1H3J	HIST1H2AI	HIST1H2BE
HIST1H4A	HIST1H2AJ	HIST1H3B
HIST1H4C	HIST1H2AK	HIST1H3C
HIST1H4D	HIST1H2AL	HIST1H3D
SCGN	HIST1H2AM	HIST1H3E
	HIST1H2BE	HIST1H3F
	HIST1H3B	HIST1H3G
	HIST1H3C	HIST1H3H
	HIST1H3D	HIST1H3I
	HIST1H3F	HIST1H3J
	HIST1H3G	HIST1H4A
	HIST1H3H	HIST1H4B
	HIST1H3I	HIST1H4C
	HIST1H4A	HIST1H4D
	HIST1H4B	HIST1H4E
	HIST1H4C	HIST1H4J
	HIST1H4D	HIST1H4K
	HIST1H4E	HIST1H4L
	HIST1H4F	
	HIST1H4J	
	HIST1H4K	
	HIST1H4L	
	MYLIP	
	RBM24	
